# The Assessment of Selected Bone and Cartilage Biomarkers in Psoriatic Patients from Poland

**DOI:** 10.1155/2015/194535

**Published:** 2015-06-04

**Authors:** Joanna Bartosińska, Anna Michalak-Stoma, Maria Juszkiewicz-Borowiec, Małgorzata Kowal, Grażyna Chodorowska

**Affiliations:** Department of Dermatology, Venereology and Pediatric Dermatology, Medical University of Lublin, 13 Radziwillowska Street, 20-080 Lublin, Poland

## Abstract

*Background*. Psoriasis is an inflammatory disease in which joints involvement may be insidious and difficult to detect. Bone and cartilage biomarkers may be helpful in screening patients with psoriasis for psoriatic arthritis (PsA). *Objectives*. To assess bone and cartilage serum biomarkers in psoriasis. *Methods*. The study was conducted in 2014 and included 61 psoriatic patients and 30 healthy individuals. In both groups, the serum concentrations of soluble receptor activator of nuclear factor-*κ*B ligand (sRANKL), cartilage oligomeric matrix protein (COMP), osteoprotegerin (OPG), and interleukin-20 (IL-20) were examined. Severity of skin lesions was assessed by Psoriasis Area and Severity Index (PASI), body surface area (BSA), and Physician Global Assessment (PGA) scores. *Results*. The duration of psoriasis was from 1 year to 45 years. 22 patients suffered from concomitant PsA. The mean value of PASI was 23.1 ± 12.0 and BSA was 27.6 ± 20.6%. COMP, OPG, and IL-20 concentrations in psoriatic patients were significantly higher than in the control group. OPG/sRANKL ratio was significantly lower in PsA patients than in psoriatic patients without arthritis. *Conclusions*. Results of the conducted study suggest that COMP, OPG, IL-20, and OPG/sRANKL ratio may appear useful biomarkers of bone and cartilage involvement in psoriasis.

## 1. Introduction

Psoriatic arthritis is a clinical manifestation of psoriasis affecting the musculoskeletal system, which according to different sources is observed in 8–10% to 30% of patients with psoriasis [1–3]. However, discrete subclinical changes in the joints and entheses may occur before the onset of PsA and may be more frequent in patients with psoriatic skin disease [4]. The PsA development may be insidious with pathological changes induced in the bones, cartilages, and tendons, which are difficult to detect since currently applied diagnostic laboratory and imaging methods are capable of recognizing only already formed abnormalities. Enthesopathy and new bone formation as well as bone loss and cartilage apposition are common in psoriatic patients [4–6]. Bones are known to be metabolically active tissues and two types of cells, osteoblasts and osteoclasts, are involved in their remodeling [7]. Local or systemic bone loss is typical for PsA leading to an increased risk of osteoporotic fractures. The increased bone resorption results from chronic inflammation and side effects induced by prolonged administration of such drugs as methotrexate and corticosteroids as well as reduced physical activity because of pain, oedema, and joint dysfunction [8]. Proinflammatory cytokines released in psoriasis stimulate formation and activation of osteoclasts which are directly responsible for the bone tissue loss [9]. They also stimulate chondrocytes to produce destructive proteases, which leads to proteoglycan loss, destruction of collagen bundles, and COMP release [5]. COMP, a homopentamer glycoprotein of the thrombospondin family, is one of the components of extracellular matrix of the joint cartilage. An elevated COMP level in the synovial fluid and serum is thought to reflect the remodeling and repair processes in the cartilage tissue [1]. Bone modeling at the entheses (enthesiophyte formation) may also occur in the absence of inflammation, but it can be the response to biomechanical stress and repeated microtraumatic injury. It can be recognized as the deep Koebner Phenomenon at the entheseal sites of psoriatic patients [4].

In PsA bone resorption, the imbalance of the RANK (receptor-activator of NF-*κ*B)/RANKL/OPG axis is thought to play a significant role. MCSF (macrophage colony-stimulating factor) and RANKL, a ligand for RANK on osteoclasts and their precursors (OCPs), are necessary for the osteoclasts formation from monocytic precursors in the synovial tissue [10]. RANK and its coreceptor, RANKL, are members of the TNF (tumor necrosis factor) superfamily. Synovial fibroblasts and activated T cells are the main source of MCSF and RANKL [11]. RANKL, a membrane protein on the osteoblasts, interacts with RANK, a type I transmembrane receptor on the bone marrow macrophages inducing their differentiation into osteoclasts [7]. It has been shown that RANKL plays a significant role in regulation of numerous functions of the dendritic cells (DCs), such as enhancement of T cell activation capacity, increased release of proinflammatory cytokines, and prolonged cell survival. Moreover, RANKL is expressed on inflamed or activated keratinocytes and it induces activation of epidermal Langerhans cells (LCs) [12].

Numerous proinflammatory cytokines, including TNF, IL-1beta, IL-6, IL-15, IL-17, and IL-23, exhibit synergic activity with RANKL in proliferation and differentiation of osteoclasts [9, 13].

Similar to MCSF and RANKL, IL-20 stimulates osteoclast differentiation. Hsu et al. [13] have shown that IL-20 increases the RANK expression on the osteoclast precursors from the bone marrow and it plays a significant role in their differentiation. Furthermore, IL-20 contributes to RANKL expression on osteoblasts.

Apart from RANKL, osteoprotegerin, a glycoprotein of the TNF family, is another receptor for RANK, which acts as a decoy receptor thereby, preventing RANKL to RANK binding and inhibiting the osteoclastogenic process. OPG is also regarded as a marker of periostitis and new bone formation [6]. The OPG/RANKL ratio is widely used to determine the bone remodeling and osteoclastogenesis [7].

Determination and monitoring of biomarkers of the bone and cartilage turnover seem to be a promising concept [5, 14]. Since the metabolites which are formed in the process of remodeling and destruction are not only present in the synovial fluid but also released into the bloodstream, they may turn out to be useful biomarkers of the bone and cartilage changes.

The aim of the study was to evaluate concentrations of selected biomarkers involved in the bone and cartilage remodeling in the serum of psoriatic patients.

## 2. Materials and Methods

### 2.1. Studied Group

The study was conducted in 2014 in patients hospitalized in the Department of Dermatology, Venereology, and Pediatric Dermatology, Medical University of Lublin, Poland, because of psoriasis exacerbation.

The study comprised 61 male psoriatic patients and 30 male healthy controls. Inclusion criteria were the duration of psoriasis for at least one year, presence of active psoriatic skin lesions on the skin, and age above 18 years old. Patients who received any systemic treatment for psoriasis within last 12 months or applied steroids, retinoids, anthralin, or vitamin D analogs on the skin within last 2 months were excluded from the study. Patients with presence of cancer, hematologic, hepatic or renal disorder, and history of musculoskeletal diseases other than PsA were excluded from the study. Twenty-two patients (36.07%) suffering from PsA met the Classification of Psoriatic Arthritis (CASPAR) criteria for PsA. All the PsA patients had polyarticular, asymmetrical subset of the disease (more than 5 joints were affected). Demographic data, medical history, blood, and serum for assessment of the selected biomarkers were collected from all the participants.

### 2.2. Assessment of Psoriasis Severity

The skin changes severity was assessed using Psoriasis Area and Severity Index (PASI), Body Surface Area (BSA), and Physician Global Assessment (PGA) scores. PASI is the most widely used tool for the measurement of severity of psoriasis. It scores the average redness, thickness, and scaling of the lesions (0–4 scale) and percentage of affected area. PASI scores range from 0 (no active disease) to 72 (the most severe disease) [14]. BSA is the percentage of Body Surface Area involved in disease. PGA is another frequently used scale, rated on a scale from clear or inactive to severe or most active [14, 15]. It is more subjective than PASI, but it is an easier and faster method to use in clinical practice [16].

### 2.3. Assessment of Cytokine Serum Concentrations in the Psoriatic Patients and Controls

Blood samples were collected from psoriatic patients and controls and were centrifuged for 15 minutes at 1000 ×g. Then, serum samples were subdivided into small aliquots to be stored at −80°C until tested for biomarkers levels. In the studied psoriatic patients as well as the control group, the concentrations of human sRANKL (total) ELISA (BioVendor, catalog number RD193004200R), human COMP (R&D Systems, catalog number DCMP0), human OPG (Demeditec Diagnostics GmbH, catalog number DE1940032), and human IL-20 (R&D Systems, catalog number DL200) were determined, according to the manufacturer's instructions. The minimum detectable dose (MDD) of sRANKL was determined to be 0.4 pmol/L. The coefficient of variation (CV) for sRANKL was 7.25–11.51% for intra-assay precision and 11.21–12.77 for interassay precision. MDD of COMP was 0.005 ng/mL. CV for COMP was 2.1–3.8% for intra-assay precision and 4.3–4.8 for interassay precision. MDD of OPG was determined to be 0.1 pmol/L. CV for OPG was 2.5–4.9 for intra-assay precision and 1.7–9.0 for interassay precision. MDD of IL-20 was 2.63 pg/mL. CV for IL-20 was 6.2–9.5 for intra-assay precision and 4.8–9.0 for interassay precision.

ESR and CPR were performed in the hospital central laboratory.

The study was approved by the Polish Local Ethics Committee.

### 2.4. Statistical Analyses

First we analysed sociodemographic and clinical data of psoriatic patients (psoriasis with and without arthritis combined, and separately psoriasis without arthritis and PsA) and control group. We used *t*-test to compare age between psoriatic patients and control group and to compare age, duration of psoriasis, PASI, and BSA between psoriatic patients without arthritis and patients with PsA. Stochastic independence *χ* ^2^ test was used to compare positive family history of psoriasis and PGA between psoriatic patients without arthritis and PsA patients.

Then, we fitted logistic regression models to explain which biomarkers have association with psoriasis and PsA. We estimated both univariate and multivariate regression models to identify biomarkers associated with psoriasis (psoriasis with and without arthritis combined) versus control group. We also fitted univariate regression models to identify biomarkers associated with PsA versus psoriasis without arthritis. Because only one biomarker was associated with PsA versus psoriasis without arthritis, there was no estimated multivariate logistic regression model. Multiple comparisons of biomarkers between the three groups (psoriasis without arthritis, PsA, and control group) were performed using polychotomous logistic regression models. The reference category was set as the control group, so we compared the effects of biomarkers in psoriatic patients without arthritis versus the control group and in PsA patients versus the control group.

As results of logistic regression analyses, we presented odds ratio (OR) that indicates change per unit increase in biomarker concentration and CI (confidence intervals) for OR. We constructed receiver operating characteristic (ROC) curves; the areas under the ROC curve were estimated to evaluate how accurately regression models classify patients into analysed groups. The cut-off values of biomarkers for diagnosis of disease were estimated as the point of intersection of curves in which both sensitivity and specificity of patients' classification for logistic regression model were the highest.

Mean values (*M*) and standard deviations (SD) were calculated for continuous variables, or absolute numbers (*n*) and relative numbers (%) of occurrence of items of categorical variables.

The Pearson correlation coefficient (*r*) was used to investigate mutual correlations between the biomarkers as well as correlation between biomarkers and clinical data in psoriatic patients.

Statistical analysis was performed using SAS System software.

In all statistical tests, significance level was set at 0.05.

## 3. Results

Sociodemographic characteristics and clinical data of psoriatic patients and control group are presented in Table 1.

The studied psoriatic patients' age was 46.4 ± 14.1 years on average and did not significantly differ with respect to control group's age (*t* = 0.654; *p* = 0.516). Psoriatic patients with and without arthritis did not differ significantly with respect to age (*t* = 0.195; *p* = 0.846). Duration of psoriasis ranged from 1 to 45 years, 20.8 ± 12.0 years on average. Duration of PsA ranged from 1 to 25 years, 10.1 ± 6.2 years on average. 21 patients (34.43%) had positive psoriatic family history.

The PASI value in the studied group ranged from 7.5 to 56.9, 23.1 ± 12.0 on average. The BSA values ranged from 4.0 to 85%, 27.6 ± 20.6% on average. The PGA score was 2 in 10 individuals (16.39%), 3 in 34 individuals (55.74%), 4 in another 12 individuals (19.67%), and 5 in 5 individuals (8.2%). Patients with and without arthritis did not differ significantly with respect to duration of psoriasis (*t* = 1.446; *p* = 0.153), positive family history (*χ* ^2^ = 0.103; *p* = 0.747), PASI (*t* = 0.912; *p* = 0.366), BSA (*t* = 0.703; *p* = 0.484), and PGA (*χ* ^2^ = 5.059; *p* = 0.168).

### 3.1. Comparing Selected Biomarkers' Serum Concentrations in the Studied Psoriatic Patients to the Control Group

**Figure 1:**
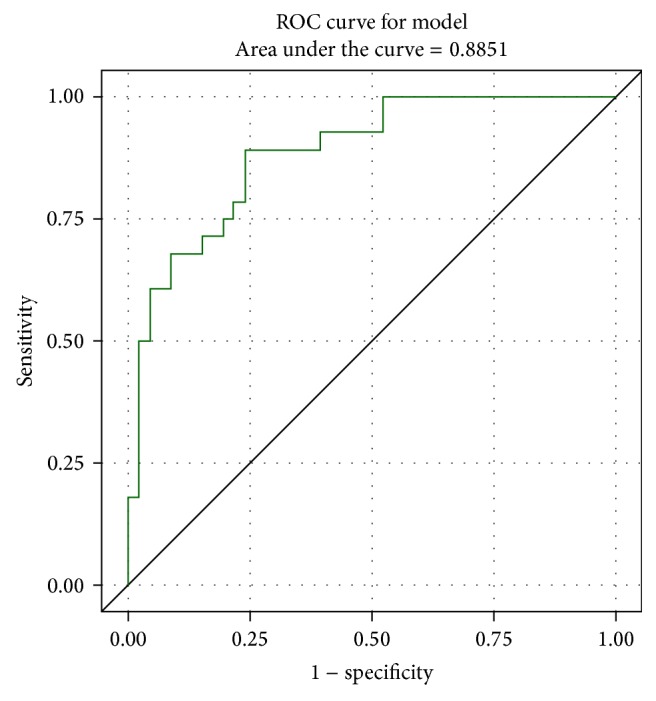
ROC curve for the reduced multivariate logistic regression model comparing patients with psoriasis and the control group.

Table 2 presents mean concentrations of the biomarkers in psoriatic patients and in the control group and the results of univariate and multivariate logistic regression analysis of psoriasis' probability. Serum concentrations of COMP, OPG, and IL-20 in the psoriatic patients were significantly higher than in the control group. Estimated odds of having psoriasis increased by about 3% if COMP increased by about 1 ng/mL, by about 25.8% if IL-20 increased by about 1 pg/mL, and almost twice if OPG increased by about 1 pmol/L. The area under the ROC curve, estimated significantly higher than 0.5, presents high accuracy of the patients' classification into patients' groups and control group based on the concentration of these three biomarkers (Figure 1).

No significant differences were found in the sRANKL and OPG/sRANKL ratio concentrations between the psoriatic patients and the control group.

ESR and CPR were within the normal ranges and there were no significant differences between the psoriatic patients and the control group as well as between psoriatic and PsA patients.

### 3.2. Comparing Selected Biomarkers' Serum Concentrations in the Psoriatic Patients without Arthritis to the PsA Patients

Table 3 presents mean concentrations of the biomarkers in the psoriatic patients without arthritis and in the PsA patients and the results of univariate logistic regression analysis of psoriasis arthritis probability. No significant differences were found in the sRANKL, COMP, OPG, and IL-20 concentrations between the psoriatic patients without arthritis and PsA patients. OPG/sRANKL ratio was significantly lower in the PsA patients than in psoriatic patients without arthritis. Estimated odds of having psoriatic arthritis decreased by about 8% if OPG/sRANKL ratio increased by about 1. The cut-off values OPG/sRANKL were estimated as 5.533 and less for diagnosis of psoriatic arthritis. It is the point of intersection of curves in which both sensitivity and specificity of patients' classification for logistic regression model were the highest.

### 3.3. Comparing Selected Biomarkers' Serum Concentrations in the Psoriatic Patients without Arthritis and PsA Patients to the Control Group

Table 4 presents the results of polychotomous logistic regression analysis of psoriasis and psoriatic arthritis probabilities. Serum concentrations of OPG and IL-20 were significantly higher both in the psoriatic patients without arthritis and in the PsA patients than in the control group (*p* = 0.05 or less). Estimated odds of having PsA and psoriasis without arthritis increased if serum concentrations of these two biomarkers increased. Serum concentration of COMP was significantly higher in psoriatic patients than in the control group (*p* = 0.007 and OR > 0), but it was not significantly different between PsA patients and the control group (*p* = 0.332).

### 3.4. Analysis of Correlations between the Determined Biomarkers' Concentrations in the Studied Psoriatic Patients

The OPG concentration was negatively correlated to psoriasis severity measured by the sPGA (*r* = −0.286; *p* = 0.031) and positively to psoriasis duration (*r* = 0.337; *p* = 0.010). It means that the lower the sPGA value and the longer the psoriasis duration were, the higher the OPG concentration was. IL-20 concentration was positively correlated to psoriasis severity expressed by PASI (*r* = 0.418; *p* = 0.001), BSA (*r* = 0.579; *p* < 0.001), and PGA (*r* = 0.392; *p* = 0.002). The higher the IL-20 concentration was, the more severe the psoriasis was on average.

No significant correlations were found between the concentrations of the other biomarkers' serum concentration and psoriasis duration, psoriatic arthritis duration, and psoriasis severity expressed by PASI, BSA, and PGA (Table 5).

In addition, mutual correlations between the concentrations of selected biomarkers in the psoriatic patients were analysed (Table 6). A significant negative correlation between sRANKL and COMP concentrations was found (*r* = −0.308; *p* = 0.044). An increase in the sRANKL was accompanied by an average decrease in the COMP concentration. No significant correlations were found between the serum concentrations of IL-20 and sRANKL, COMP, and OPG as well as between OPG and sRANKL and COMP.

## 4. Discussion

Gladman et al. [17] have observed that 16% of the patients with a 14-year-long psoriasis history developed deformations in more than five joints. Early diagnosis of PsA (i.e., before its clinical manifestations or irreversible changes have developed) is essential since prompt medical intervention is likely to stop the progress of the disease and prevent joint destruction. Therefore, sensitive and specific diagnostic tools are still in demand. In the light of recent studies, patients with isolated cutaneous psoriasis may have subclinical periarticular bone changes in a form of new bone formation in the entheseal regions of the joints (enthesiophytes) [4].

In our own study significantly higher COMP, OPG, and IL-20 serum concentrations in psoriatic patients were found in comparison to the control group; the results were independent of the presence of PsA. This could imply that, in some of the studied psoriatic patients without arthritis, asymptomatic osteoarticular changes are being formed. Hence, in order to prevent formation of irreversible changes and slow down the progress of the disease, close monitoring of such patients and early intervention should be executed.

In the cartilage, made up of chondrocytes imbedded in the matrix, proteoglycans and type II collagen are the fundamental constituents. Activation of cytokines (IL-1, IL-17, TNF, and oncostatins) stimulates chondrocytes to release destructive proteases, which will result in the proteoglycan loss and destruction of collagen bundles. Consequently, cartilage macromolecules, such as aggrecan and COMP, are released into the synovial fluid and serum, which may be reflective of the cartilage turnover [1].

The studies conducted so far indicated that in patients with PsA the level of COMP in the synovial fluid and serum is increased [1, 18]. COMP expression is confirmed not only in the cartilage but also in tendons, entheses, and ligaments as well. Therefore, COMP expression may be regarded as an early marker of cartilage destruction and turnover in the PsA patients [5].

Månsson et al. [1] have shown that COMP concentration in the synovial fluid is significantly higher in PsA than rheumatoid arthritis (RA). This, according to the authors, may be related to the degradation and release of newly synthesised molecules reflecting a repair process. Furthermore, this repair process appears to be sufficient for preventing permanent cartilage destruction [1].

The study results concerning correlation of COMP and PsA activity are inconclusive. According to some researchers, serum COMP correlates with PsA activity as well as with inflammation indicators such as CRP, ESR (erythrocyte sedimentation rate), and the number of oedemic joints [18]. Thus, serum COMP could reflect the PsA activity. However, Månsson et al. [1], similar to our findings, did not reveal any correlation between COMP concentration and levels of inflammatory indicators.

There are reports which present COMP as a biomarker in psoriatic patients without arthritis. Skoumal et al. [18] found out that the severity of skin lesions had no effect on the level of serum COMP which depended only on the coexisting inflammatory changes in the joints. Their patients with active PsA presented markedly elevated serum COMP levels in comparison to the PsA patients with low clinical and laboratory activity of the disease. The results of ultrasound (US) examination in a group of 60 psoriatic patients (30 with psoriasis without arthritis and 30 with PsA) revealed that the prevalence of entheseal abnormalities was not significantly different between psoriatic patients without arthritis and PsA patients (*p* = 0.19) [5]. Therefore, according to Farouk et al. [5], the level of sCOMP is a more sensitive biomarker, whereas the ultrasound is more specific. In both studied groups, a correlation was found between COMP and CRP as well as between COMP and DAS28. However, no correlation with PASI was detected. On the basis of performed US, the authors concluded that enthesitis may be asymptomatic and some cases of Achilles tendon enthesopathy remain undetected/undiagnosed. The elevated COMP levels in the psoriatic patients without arthritis and PsA patients suggest that undetected articular involvement may also be present in psoriatic patients without arthritis [5]. All these findings give grounds for close monitoring of psoriatic patients without arthritis with elevated sCOMP levels so that further PsA development could be prevented.

The RANK-RANKL-OPG axis may be another group of useful biomarkers in psoriasis. OPG, also known as the osteoclast differentiation-inhibiting protein, is an important osteoclastogenesis inhibitor. The studies conducted so far confirm the role of RANKL-promoted osteoclastogenesis in the PsA bone resorption and osteoporotic fractures [7]. The increased risk of developing osteoporosis by psoriatic patients is largely connected with an ongoing chronic inflammatory process [9]. In our study, OPG concentration was significantly higher in psoriatic than control group and was positively correlated with psoriasis duration; the longer the disease duration, the higher the OPG concentration. However, the negative correlation among OPG concentration and sPGA was noted.

The pathogenetic correlation between the immune system and bone metabolism is well established and OPG and RANKL are known to play a role in the activation of T cells, chronic inflammation, and bone resorption. Activated T cells release RANKL which favours the pathogenesis of inflammatory bone diseases and bone loss. The cytokines released in the inflammatory process stimulate production and activation of the osteoclasts, whereas OPG, as a decoy receptor, increases bone density [8].

In Hofbauer et al. [8]'s study, the serum OPG level was not significantly different between the PsA patients and the control group. Interestingly, PsA females had higher OPG concentrations in comparison with the male patients, which may be explained by the stimulatory effect of estrogens on OPG. The authors' opinion was that low osteoporosis prevalence in PsA women may be explained by the compensatory mechanism of the elevated OPG level [8].

Unfortunately, the positive effect of the elevated OPG in psoriasis is not sufficient to prevent further osteopathogenic processes. Ritchlin et al. [19] have found that osteoclast precursors are markedly increased in the serum of PsA patients, especially in those with bone erosions confirmed by the radiograph. Moreover, in their immunohistochemical examinations, the authors confirmed RANKL expression on the synovial lining cells, while OPG expression was limited to the endothelium. Because of this, OCPs with RANK receptor are likely to induce osteoclastogenesis in the synovial environment, where high RANKL/OPG ratio is present. The RANKL-RANK interaction results in OCPs differentiation into multinuclear bone-resorbing osteoclasts. Numerous cytokines, including the IL-23/IL-17 axis, increase RANK expression. Also IL-20, released by monocytes, keratinocytes, and Th17 cells and IL-20R in the synovium of PsA patients, increases RANK and RANKL expression [20]. In our study, IL-20 was significantly higher in both psoriatic patients without arthritis and PsA patients in comparison with the control group. However, no difference in IL-20 concentration was found among psoriatic patients without arthritis and PsA patients. Hsu et al. [13] have pointed out that IL-20 is higher in patients with bone loss resulting from osteoporosis. IL-20 stimulates differentiation of osteoclasts; therefore, blocking of this cytokine by monoclonal antibodies may be of therapeutic significance. M-CSF and RANKL are known to be indispensible and sufficient for osteoclasts differentiation. The authors have shown that IL-20 increased RANK expression on the osteoclasts precursors from bone marrow. In addition, IL-20 also increased RANKL expression on osteoblasts. TNF also promoted the formation of osteoclasts, and infliximab application prevented bone loss in PsA patients [8].

Toberer et al. [12] found a significantly increased RANKL expression in psoriatic epidermis, while it was not observed in the patients with cutaneous lupus erythematosus. This could be explained by Treg functional insufficiency in psoriatic skin lesions that resulted in decreased effector T cell inhibition.

Previous reports suggest that RANKL may be helpful in identification of patients with progressive arthritis or in those with an aggravated course of the disease [6]. In our study, maybe because of a less severe course of PsA (ESR and CPR were within the normal ranges), sRANKL was not significantly different among psoriatic patients in comparison with the control group.

Attia et al. [9] observed significantly increased OPG serum levels in both psoriatic patients without arthritis and PsA patients suggestive of the presence of osteoporosis regardless of the sex, age, BSA, and PASI. They also found a correlation between the number of affected joints and an increased risk of developing osteoporosis.

Therefore, apart from determination of the concentration of individual biomarkers, it is essential that the OPG/RANKL ratio should be calculated. Xue et al. [7], who made an attempt to determine selected osteoclastogenesis biomarkers, revealed that the concentrations of TNF-alfa, RANKL, and osteoclast precursors were significantly higher in PsA patients than in those with psoriatic patients without arthritis and the control group. The OPG/RANKL ratio was significantly lower in PsA than in psoriatic patients without arthritis. No statistically significant difference in the OPG concentrations between the controls and PsA patients was found, and neither was it found between the patients with erosive and nonerosive PsA. They also found a positive correlation between RANKL and osteoclast precursors' concentrations as well as PsAJAI (Psoriatic Arthritis Joint Activity Index), which further confirms the bone remodeling and RANKL/osteoclastogenesis activity in PsA [7]. Having observed significantly higher values of the OPG/RANKL ratio in psoriatic patients without arthritis in comparison with the controls, the same authors suspected the presence of unidentified factors capable of preventing osteoclastogenesis in psoriatic patients without arthritis. Interestingly, the study results presented by Xue et al. [7] are consistent with the results obtained in our study, where the OPG/sRANKL ratio was significantly higher in the psoriatic patients without arthritis than in PsA patients (*p* = 0.013). However, the fact that we have determined the levels of biomarkers merely in the serum of psoriatic patients may be a limitation of our study and, therefore, further studies are needed to be performed in this area, for example, simultaneous determination of the cartilage and bone biomarkers in the synovial fluid and psoriatic skin specimens.

It seems likely that determination of the cartilage and bone remodeling biomarkers in psoriatic patients will make it possible to identify the patients with an increased risk of developing PsA. Determination of more than one biomarker appears to increase the possibility of assessing not only severity but also activity of the disease and identification of both destructive and new bone formation pathogenic processes. Thus, to define the prognostic role of biomarkers in psoriasis still remains a challenge.

## 5. Conclusions

1. Results of the conducted studies suggest that COMP, osteoprotegerin, interleukin-20, and osteoprotegerin/sRANKL ratio may appear useful biomarkers of bone and cartilage involvement in psoriasis.
2. The serum concentrations of COMP, osteoprotegerin, and IL-20 were increased in the psoriatic patients.
3. The studied biomarkers' concentrations were not significantly different in psoriatic patients without arthritis and psoriatic arthritis patients.
4. Osteoprotegerin/sRANKL ratio was significantly lower in the psoriatic arthritis than in psoriatic patients without arthritis.
5. Some bone and cartilage biomarkers correlated with psoriasis severity and its duration.

## Figures and Tables

**Table 1:** Sociodemographic characteristics and clinical data of psoriasis patients and control group.

Characteristics	Psoriasis			Control (*N* = 30)	*p*	
	Total (*N* = 61)	Arthritis (*N* = 22)	Without arthritis (*N* = 39)		Psoriasis versus control	Psoriatic arthritis versus psoriasis without arthritis
Age (years), M ± SD	46.4 ± 14.1	48.0 ± 10.6	45.5 ± 15.8	45.7 ± 11.3	0.516	0.846
Sex-male, *n*; %	61; 100.00	22; 100.00	39; 100.00	30; 100.00	—	—
Duration of psoriasis (years), M ± SD	20.8 ± 12.0	23.7 ± 9.0	19.1 ± 13.3	—	—	0.153
Duration of psoriatic arthritis (years), M ± SD	—	10.1 ± 6.2	—	—	—	
Positive family history of psoriasis, *n*; %	21; 34.43	7; 31.82	14; 35.89	—	—	0.747
PASI, M ± SD	23.1 ± 12.0	21.3 ± 9.1	24.2 ± 13.4	—	—	0.366
BSA (%), M ± SD	27.6 ± 20.6	25.1 ± 15.6	29.0 ± 23.0	—	—	0.484
PGA, *n*, %				—	—	0.168
2	10; 16.39	2; 9.09	8; 20.51	—		
3	34; 55.74	15; 68.18	19; 48.72	—		
4	12; 19.67	5; 22.73	7; 17.95	—		
5	5; 8.20	0; 0.00	5; 12.82	—		

**Table 2:** The biomarkers serum concentrations in the psoriatic patients compared to the control group.

Biomarker	Psoriasis	Control	Univariate logistic regression analysis (psoriasis versus control)		Multivariate logistic regression analysis (psoriasis versus control)	
	M ± SD	M ± SD	OR (95% CI)	*p*	OR (95% CI)	*p*
sRANKL (pmol/L)	94.589 ± 74.330	87.235 ± 67.028	1.003 (0.996, 1.011)	0.357	—	—
COMP (ng/mL)	592.111 ± 337.873	417.388 ± 215.801	1.002 (1.000, 1.004)	0.020	1.003 (1.000, 1.005)	0.027
Osteoprotegerin (pmol/L)	3.682 ± 1.270	3.070 ± 0.741	1.823 (1.073, 3.098)	0.027	1.993 (1.001, 4.156)	0.048
Osteoprotegerin/sRANKL	9.104 ± 9.317	14.801 ± 21.747	1.025 (0.985, 1.066)	0.223	—	—
IL-20 (pg/mL)	24.052 ± 17.965	7.517 ± 4.632	1.232 (1.116, 1.360)	<0.001	1.258 (1.115, 1.420)	<0.001

**Table 3:** The biomarkers serum concentrations in the psoriatic patients with and without arthritis.

Biomarker	Psoriasis without arthritis	Psoriatic Arthritis	Logistic regression analysis (psoriatic arthritis versus psoriasis without arthritis)	
	M ± SD	M ± SD	OR (95% CI)	*p*
sRANKL (pmol/L)	85.242 ± 71.819	110.947 ± 77.652	1.005 (0.997, 1.012)	0.221
COMP (ng/mL)	646.779 ± 343.413	495.638 ± 314.577	0.999 (0.997, 1.000)	0.145
Osteoprotegerin (pmol/L)	3.684 ± 1.380	3.678 ± 1.088	0.996 (0.649, 1.528)	0.986
Osteoprotegerin/sRANKL	19.421 ± 25.687	6.177 ± 4.897	0.920 (0.843, 0.998)	0.049
IL-20 (pg/mL)	25.928 ± 19.222	20.746 ± 15.388	0.982 (0.948, 1.016)	0.297

**Table 4:** Logistic regression analyses of the biomarkers serum concentrations in the psoriasis without arthritis and psoriatic arthritis compared to the control group.

Biomarker	Analysis of effects	Psoriasis without arthritis versus control		Psoriatic arthritis versus control	
	*p*	OR (95% CI)	*p*	OR (95% CI)	*p*
sRANKL (pmol/L)	0.277	1.001 (0.993, 1.010)	0.736	1.007 (0.998, 1.015)	0.138
COMP (ng/mL)	0.022	1.003 (1.001, 1.005)	0.007	1.001 (0.999, 1.003)	0.332
Osteoprotegerin (pmol/L)	0.085	1.825 (1.049, 3.178)	0.033	1.818 (0.999, 3.307)	0.050
Osteoprotegerin/sRANKL	0.057	1.043 (0.996, 1.092)	0.074	0.956 (0.876, 1.044)	0.319
IL-20 (pg/mL)	<0.001	1.241 (1.123, 1.372)	<0.001	1.218 (1.101, 1.348)	<0.001

**Table 5:** Correlation coefficients between the biomarkers serum concentrations and clinical data in the psoriatic patients.

Biomarker	Duration of psoriasis (years)	Duration of psoriatic arthritis (years)	PASI	BSA (%)	PGA
sRANKL (pmol/L)					
*r*	−0.146	−0.337	0.077	−0.018	0.020
*p*	0.289	0.147	0.578	0.894	0.885
COMP (ng/mL)					
*r*	0.077	−0.152	−0.193	−0.073	−0.069
*p*	0.607	0.561	0.193	0.624	0.646
Osteoprotegerin (pmol/L)					
*r*	**0.337**	0.126	−0.208	−0.224	**−0.286**
*p*	**0.010**	0.587	0.121	0.093	**0.031**
Osteoprotegerin/sRANKL					
*r*	0.284	0.013	−0.223	−0.131	−0.144
*p*	0.124	0.962	0.150	0.401	0.358
IL-20 (pg/mL)					
*r*	−0.051	−0.145	**0.418**	**0.579**	**0.392**
*p*	0.703	0.532	**0.001**	**<0.001**	**0.002**

**Table 6:** Mutual correlation coefficients between the biomarkers serum concentrations in the psoriatic patients.

Biomarker	COMP (ng/mL)	Osteoprotegerin (pmol/L)	IL-20 (pg/mL)
sRANKL (pmol/L)			
*r*	**−0.308**	−0.018	0.177
*p*	**0.044**	0.905	0.200
COMP (ng/mL)			
*r*	—	0.178	0.022
*p*	—	0.236	0.886
Osteoprotegerin (pmol/L)			
*r*	—	—	−0.019
*p*	—	—	0.887
